# Prevalence of maladaptive daydreaming among medical students at the University of Khartoum, Sudan, in 2020–2021

**DOI:** 10.1186/s43045-021-00122-8

**Published:** 2021-08-10

**Authors:** Moez Mohammed Ibrahim Bashir

**Affiliations:** University of Khartoum, P.O. Box 103, ElQasr Ave, Khartoum, Khartoum Sudan

**Keywords:** Maladaptive daydreaming, Maladaptive daydreaming among medical students, Medical students’ mental health, Mental health in Sudan, Psychiatry in Sudan

## Abstract

**Background:**

Daydreaming is a normal cognitive phenomenon in which the individual experiences a temporary separation from reality during which contact with reality is compromised and, to some extent, replaced by a visionary fantasy. On the other hand, daydreaming can progress to a maladaptive state, known as *maladaptive daydreaming*. The aim of this study was to help understand the full scope of *maladaptive daydreaming* among medical students at the University of Khartoum, in Sudan, by displaying all *maladaptive daydreaming*-related statistics among studied population. An analytical cross-sectional university-based study was conducted during the academic year 2020–2021, using a total of 323 self-administered online questionnaires; the questionnaire containing the Arabic Version of Maladaptive Daydreaming Scale and other sociodemographic data were given by stratified random selection to undergraduate medical students at the University of Khartoum, in Sudan, between December and January 2021.

**Results:**

34.3% of medical students at the University of Khartoum have identified themselves as maladaptive daydreamers. More males were found to be maladaptive daydreamers when compared to female medical students. The average Maladaptive Daydreaming Scale score among medical students was found to be 33.6 (sd=22.3). No significant correlation was found between Maladaptive Daydreaming Scale score and gender, and there was a significant correlation between the MDS score and the academic class of medical students at University of Khartoum.

**Conclusions:**

*Maladaptive daydreaming* was discovered to have a significant impact on the population of medical students at the University of Khartoum, with some alarming rates and correlations among medical students; having more than one-third of total medical students found to be having MD must surely indicate a much larger and more widespread problem. A multifactorial holistic approach covering biological, psychological, social, and academic aspects must be used when hypothesizing the justification for these findings and planning an active intervention strategy.

## Background

Daydreaming, also known as spacing out, is a normal cognitive phenomenon in which the individual experiences a temporary separation from reality during which contact with reality is compromised and, to some extent, replaced by a visionary fantasy. However, daydreaming can progress to a pathological or maladaptive state, known as maladaptive daydreaming (MD), which is defined as “extensive fantasy activity that replaces human interaction and/or interferes with academic, interpersonal, or vocational functioning” [1–3]. Daydreaming for extended periods of time, extremely vivid dreams, difficulty executing and completing daily tasks, displaying some facial expressions, performing repetitive movements, and whispering or talking while daydreaming are all common manifestations of maladaptive daydreaming [4]. Maladaptive daydreaming is an under-researched issue with a remarkable growing interest among psychiatrists, psychologists, and health educators due to its grave comorbidities and serious impact on the daily life performance of maladaptive daydreamers in the general population, particularly medical students [5, 6]. Up until this date, MD does not meet any existing mental disorder diagnosis criteria but overlaps with several psychopathological conditions. In terms of psychiatric comorbidity, 74.4% of people with MD meet the criteria for more than three psychiatric disorders, while 41.1% meet the criteria for more than four psychiatric disorders, ADHD was the most common comorbid disorder (76.9%); 71.8 percent met criteria for an anxiety disorder, 66.7 percent for a depressive disorder, and 53.9 percent for an obsessive-compulsive or related disorder [5]. It has also been indicated that people with MD had more pronounced symptoms of obsessive-compulsive disorder, generalized and social anxiety, dissociation, more negative emotions, and a lower expression of positive emotions [7]. According to a recent study conducted in Saudi Arabia, 70% of medical students engage in maladaptive daydreaming. Furthermore, maladaptive daydreaming has been linked to poor academic performance, with academic performance significantly lower among maladaptive daydreamers (MDers) compared to non-MDers, highlighting the seriousness of the condition among medical students [8]. The knowledge gap of maladaptive daydreaming-related statistics among medical students, or rather, in Sudan as a whole, is unmistakably large, which is a very concerning fact given the magnitude of this phenomenon. This study was carried out to narrow the knowledge gap of maladaptive daydreaming-related statistics, specifically on medical students at the University of Khartoum, in order to create a starting point for future studies on maladaptive daydreaming, exploring its prevalence, related medical and psychiatric conditions, and its impact on maladaptive daydreamers.

## Methods

### Study design

This was an analytical cross-sectional university-based study that was conducted during the academic year 2020–2021. The research was carried out virtually at the Faculty of Medicine at the University of Khartoum in Khartoum, Sudan.

### Study area

The research was carried out virtually at the University of Khartoum’s Faculty of Medicine (UofK-MED). UofK-MED is Sudan’s oldest medical school, having been founded in 1924. It was also the first medical school in Sudan to be accredited by the World Federation of Medical Education. Every year, thousands of students apply for admission to UofK-MED, attracted by its unique medical education and extracurricular opportunities.

### Study population

During the academic year 2020–2021, there are 2335 students enrolled at the college. All of the participants were undergraduate medical students, with the majority of them ranging in age from 18 to 23. In terms of inclusion criteria, all undergraduate students at the University of Khartoum–Faculty of Medicine in Khartoum in the first (batch 96), second (batch 95), third (batch 94), fourth (batch 93), fifth (batch 92), and sixth (batch 91) academic years were included in the study, and no students were excluded.

### Sample size and sampling

A random sample of 323 students was chosen from the medical students at the University of Khartoum, Faculty of Medicine, using stratified random sampling. Sample size (n) was calculated according to the formula: n = [z2 * p * (1−p)/e2]/[1 + (z2 * p * (1−p)/(e2 * N))] where z = 1.96 for a confidence level (α) of 95%, p is the proportion (expressed as a decimal), N is the population size, and e is the margin of error.

$$ \mathrm{z}=1.96,\mathrm{p}=0.5,\mathrm{N}=1994,\mathrm{e}=0.05 $$

$$ \mathrm{n}=\left[1.962\ast 0.5\ast \left(1--0.5\right)/0.052\right]/\left[1+\left(1.962\ast 0.5\ast \left(1-0.5\right)/\left(0.052\ast 1994\right)\right)\right] $$

$$ \mathrm{n}\approx 323 $$

The sample size (with finite population correction) is equal to 323.

The study population was subdivided into 6 groups according to their academic classes, and list containing all students in each academic class was obtained. Since the study sample size was 323, a proportionate random sample from each group was selected using the online sample randomizer at https://www.randomizer.org.

Fifty-two out of 325 were selected from the sixth-year students (batch 91), 51 out of 313 from the fifth-year students (batch 92), 56 out of 349 from the fourth-year students (batch 93), 58 out of 358 from the third-year students (batch 94), 54 out of 333 from the second-year students (batch 95), and 52 out of 326 from the first-year students (batch96).

### Data collection methods and tools

During the academic year, all targeted students completed online self-administered closed-ended questionnaires. Before filling out the Arabic version of the Maladaptive Daydreaming Scale, participants were asked demographic and general information questions: age, gender, marital status, and academic class. Each individual was approached separately, either directly using their personal social media accounts, or through the students’ representing bodies within UofK-MED; this process proved to be effective, yielding a 100% response rate.

The Arabic Version of the Maladaptive Daydreaming Scale (MDS-16-AR) is a new 16-item scale proposed and validated in 2019 by Hisham Motkal Abu Raya, Professor Eli Somer, and Suha Meari-Amir [7]. It consists of 16 items that assess some of the most important aspects of MD. The scale responses range from 0 to 100%, with 10% intervals (0% = never/never; 100% = all of the time/extreme amounts). The MDS-16-AR was chosen as the measurement tool for MD in this study not only because it uses the research subjects’ mother tongue, but also because it demonstrated high sensitivity (89), specificity (87), and overall validity (85.5) with a cutoff composite score of 45 (out of a maximum of 100) [7, 9, 10].

### Data management and statistical analysis

Data was entered and encoded then analyzed by the computer programs by using the SPSS software (version 21). The collected data was analyzed through descriptive analysis and using the chi square and t-tests. P value <0.05 was considered statistically significant. Data were displayed and presented in form of tables.

## Results

**Fig. 1:**
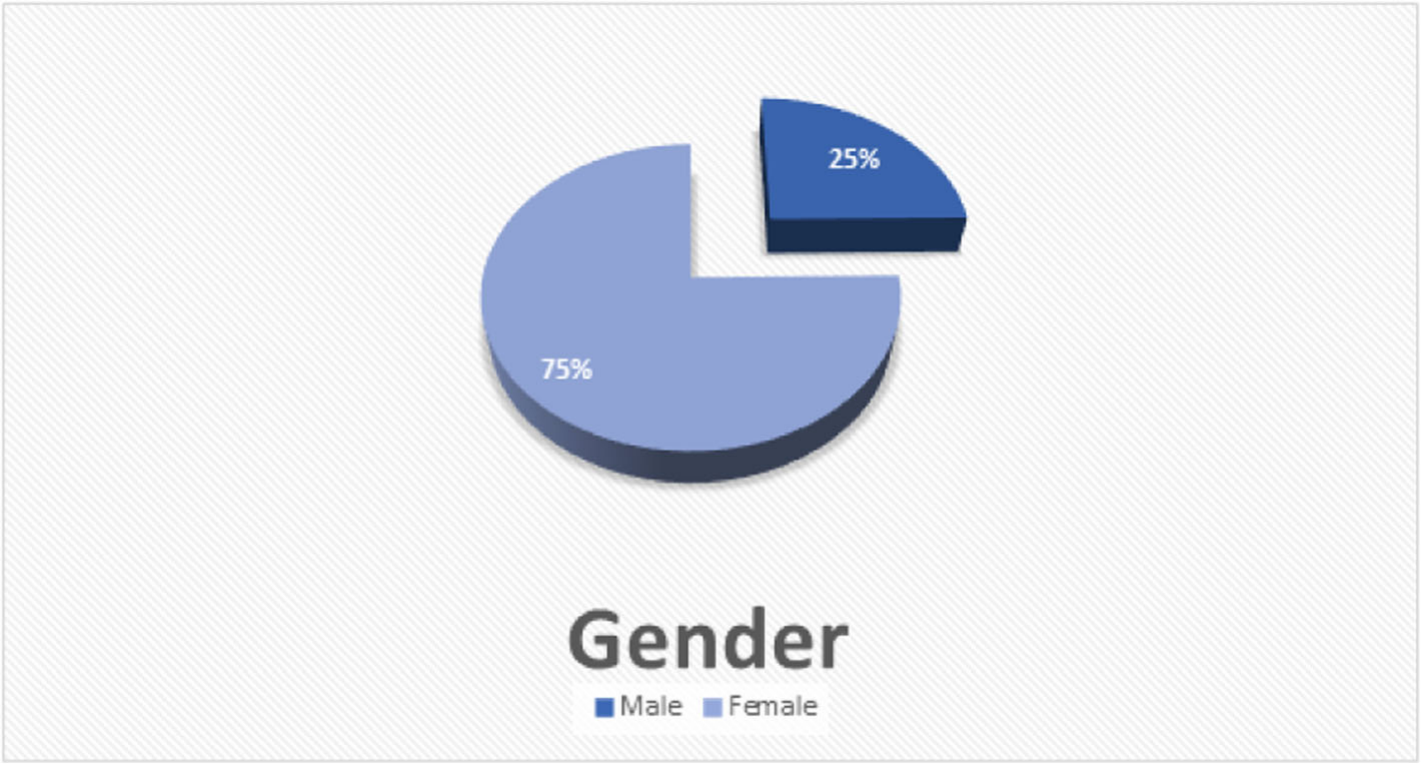
Gender. The percentage of males and females who participated in the study at University of Khartoum 2020–2021. The males were 80 (25%) and the females were 243 (75%)

The studied sample in this study was *323* medical students; all 323 students were given the study questionnaire and *response rate of which was 100%*. Sixth-year students represented 16.1% of the sample, fifth-years represented 15.8%, fourth-years represented 17.3%, third-years represented 18%, second-years represented 16.7%, and first-years represented *16.1* students. Twenty-five percent of the studied population were *males*, while the *females* made *75%* out of the total study sample (Fig. 1). All of the studied students were unmarried.

**Fig. 2:**
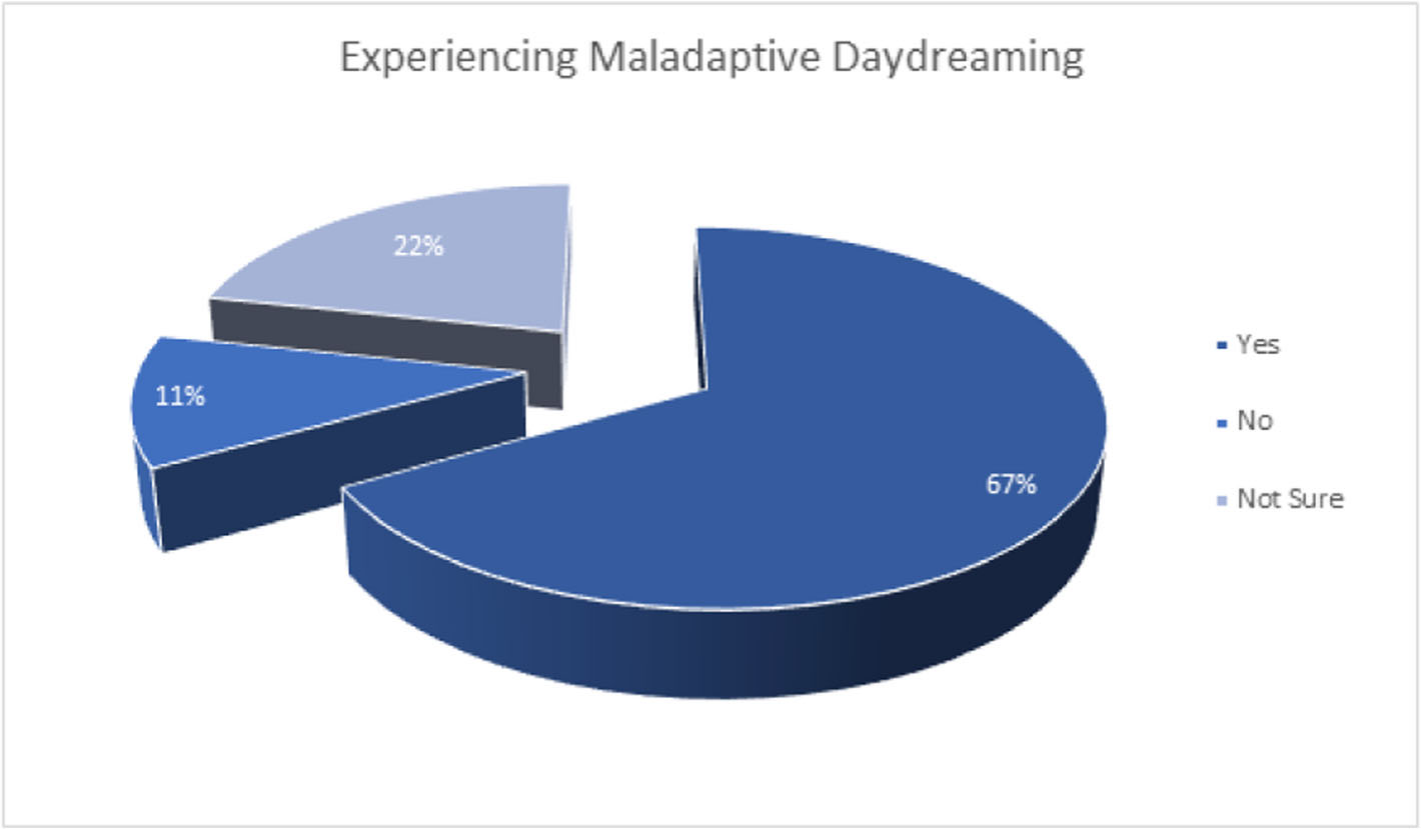
Experiencing maladaptive daydreaming. The percentage of experiencing daydreaming among the research participants. Sixty-seven percent of the study participants have experienced daydreaming, while 11% did not. Twenty-two percent remained unsure whether or not they have experienced daydreaming

**Fig. 3:**
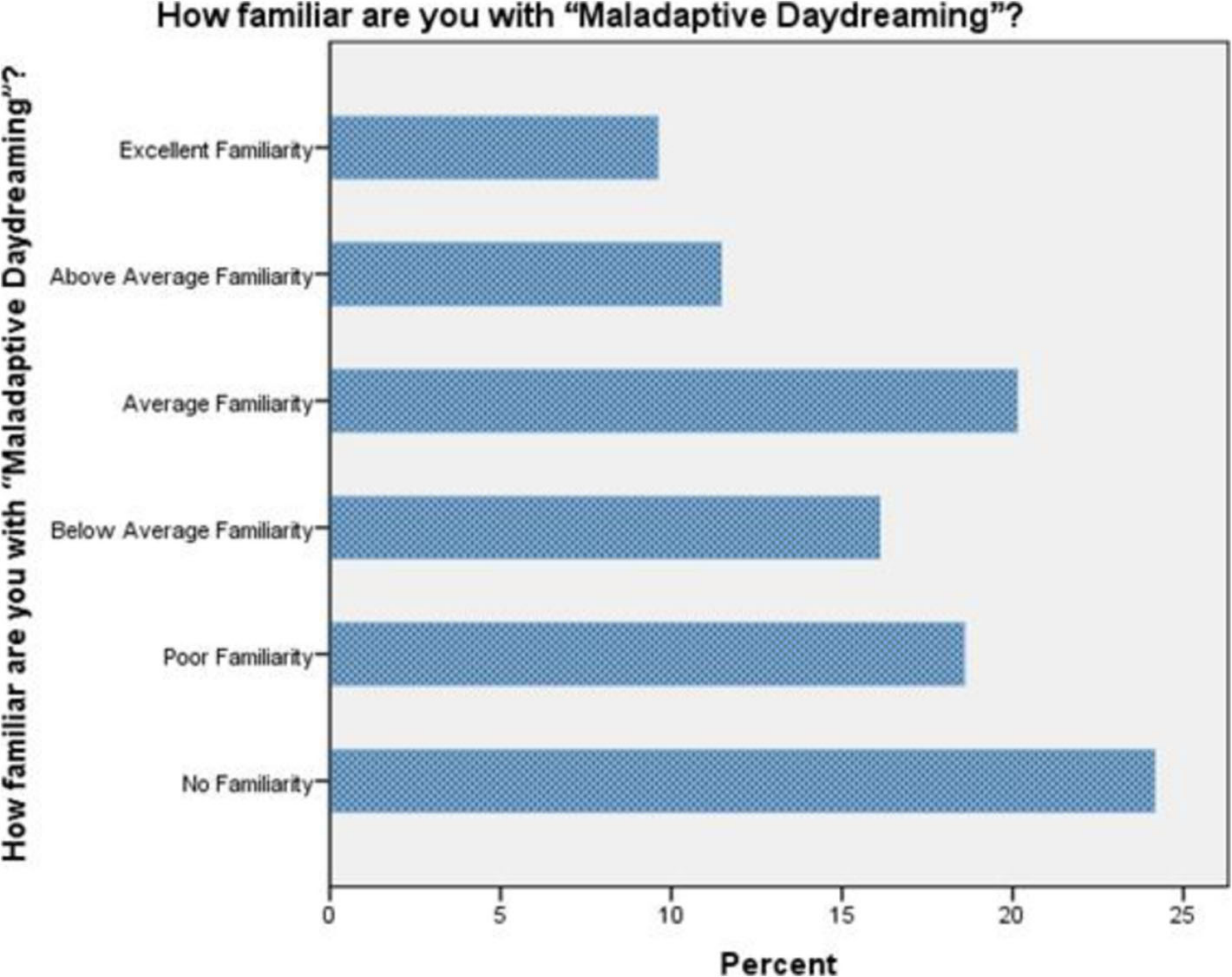
Being familiar with the term maladaptive daydreaming. The percentage of maladaptive daydreaming familiarity among the study participants. 24.1% had no familiarity with the concept of maladaptive daydreaming, 18.6% had poor familiarity, 16.1% had below average familiarity, 20.1% had average familiarity, 11.5% had above average familiarity, and 9.6% had excellent familiarity

Sixty-seven percent of the study participants reported that *they have experienced daydreaming*, while 11% *said they have not*, while 22% *remained unsure* whether or not they have experienced daydreaming (Fig. 2), 62.5% of male reported experiencing daydreaming, while 68.3% of females reported the same finding (Table 1). The correlation between gender and experiencing daydreaming among study participants was tested using the Chi-Square tests, and *no significant correlation was found between gender and experiencing normal daydreaming* (P=0.179). When asked about their subjective familiarity with the concept of MD “*How familiar are you with the term Maladaptive Daydreaming?*”, 24.1% had *no familiarity with the concept at all*, 18.6% had *poor familiarity*, 16.1% had *below average familiarity*, 20.1% had *average familiarity*, 11.5% had *above average familiarity*, and 9.6% had *excellent familiarity* (Fig. 3). When asked about whether or not they feel their daydreaming activities interfere with their academic performance, *38.8% of students felt that there was no kind interference whatsoever*, while 5.3% felt *maximum interference*, the rest fell in between.

**Table 1:** Males and females experiencing daydreaming

Experiencing daydreaming?		Yes	No	Not Sure	Total
**Male**	N	50	7	23	80
**Female**	N	166	30	47	243
**Total**	N	216	37	70	323

The percentage of male and female study participants who reported experiencing daydreaming in their life. 62.5% of male reported experiencing daydreaming, while 68.3% of females reported the same finding

**Fig. 4:**
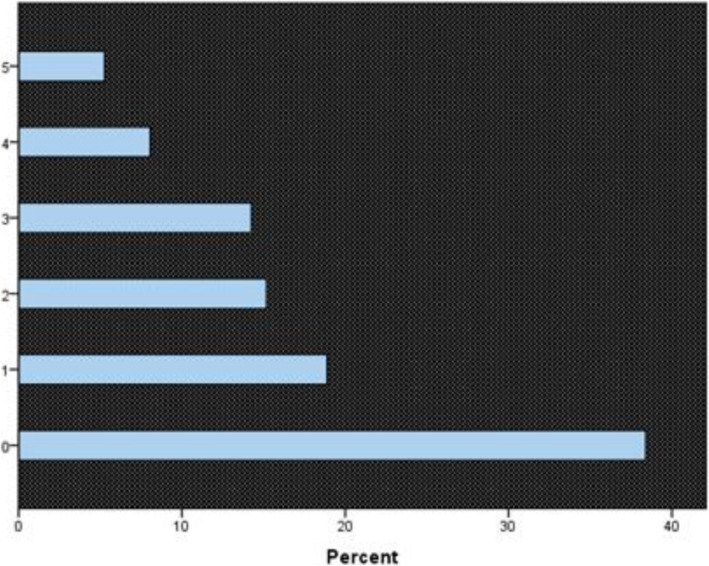
Interference with academic performance. Shows how much students feel that their daydreaming activities interfere with achieving their overall academic performance. 38.8% of students feel that there was 0 interference while 5.3% felt maximum interference

MDS score was found to be 87.14 with a minimum score of 0 and a maximum 87.14. The mean was found to be 33.66. The prevalence of MD (MDS score ≥ 45) among medical students at University of Khartoum (n=323) was 34.3% (CI 95%). *37.8% of males were found to be MDers* and *33.3% of females were MDers* (Table 2); the correlation between gender and MDS score was tested using Pearson’s correlation test, and no significant correlation was found (P=0.072). The percentage of MDers in each academic class was *13.4% in sixth class*, *15.9% in fifth class students*, *35.7% in fourth class*, *51.7% in third class*, *46.3% in second class*, and *40.3% among first year* medical students at the University of Khartoum (Table 3). A significant correlation was found between academic class and MDS score (P=0.005). When asked whether or not they feel that MD has interfered with their academic functioning, 38.8% of total students believed there was no interference, 61.2% believe MD has affected their academic performance to a limited extent, and 5.3% reported the greatest amount of interference (Fig. 4).

**Table 2:** Maladaptive daydreaming valid cases, by gender

Male	N	Valid cases	30
		Total Males	80
Female	N	Valid cases	81
		Total Females	243
**Total cases**	**111**		
**Total sample**	**323**		

The percentage of MDers among medical students at University of Khartoum. 34.3% of total students were found to be MDers. 37.8% of males were MDers and 33.3% of females were MDers

**Table 3:** Maladaptive daydreaming valid cases by academic class

Sixth	N	Valid cases	7
		Total sample	52
		**Percentage**	**13.4%**
Fifth	N	Valid cases	8
		Total sample	51
		**Percentage**	**15.9%**
Fourth	N	Valid cases	20
		Total sample	56
		**Percentage**	**35.7%**
Third	N	Valid cases	30
		Total sample	58
		**Percentage**	**51.7%**
Second	N	Valid	25
		Total	54
		**Percentage**	**46.3%**
First	N	Valid	21
		Total	52
		**Percentage**	**40.3%**

Percentage of MDers in each academic class, 13.4% of sixth class, 15.9% of fifth class students, 35.7% of fourth class, 51.7% of third class, 46.3% of second class, and 40.3% of first year medical students at the University of Khartoum were MDers

## Discussion

This study represents the first Sudanese study of maladaptive daydreaming, the aim of which was to investigate the full scope of MD among medical students at the University of Khartoum in Sudan, including the prevalences of daydreaming, MD among all medical students, and MD among each academic class students in separate. The study also investigated the association between MD and other factors. Since most of the college students at UofK-MED are females. In their responses, only 67% of the study participants have experienced daydreaming, which is far less than the percentage of daydreaming among the American population (96%) [2, 3]. The difference between the two percentages might be explained by many factors; a clear explanation is the wide cultural differences between the Sudanese and the American population; another possible explanation is the presumable lack of sufficient knowledge of the concept and the experience of maladaptive daydreaming among the study population, which is a plausible explanation because 24.1% of the study participants were not familiar with the concept of maladaptive daydreaming. One important finding regarding the experience of normal daydreaming is that there was no significant correlation between daydreaming and gender of medical students at the UofK-MED (P=0.179); the same finding was found in many similar studies [5, 6, 8]

Regarding the MDS score, the mean of MDS score among the study population was found to be 33.66, which is still considered less than the 45% cutoff point measurement for MD. As mentioned in the “Results” section, *34.3%* of total students were found to be MDers; on the other hand, 70% of the of students were found to be MDers in a similar study conducted on Saudi medical students [8], while in Italy 17% of adult Italian population during the COVID-19 lockdown were identified as MDers [11]; again, this could be explained by cultural, socioeconomic, and demographic differences between the countries. Additionally, the two abovementioned studies was conducted on 380 students chosen by convenient sampling, whereas this study population was chosen by random sampling. In this study, although no significant correlation was found between gender and MDS score, in contrast to other studies in which age was found to be negatively associated with MD [6], more males were found to be MDers when compared to females, which supports the findings of multiple similar studies [4–6]. Since a significant correlation between MDS score and academic class of study subjects, we noticed that the MDS score hugely varies in each academic class. Third-year medical students were non-arguably the most alarming, since more than half of the students were identified as MD. The study also demonstrates the deep and concerning perception of medical students at the University of Khartoum regarding the impact of MD on their academic functioning and also emphasizes the academic gravity of the situation, as MD clearly has a negative impact on medical students’ academic achievement over 60%; in fact, a significant correlation was found between MDS score and medical students’ perception of its negative impact on their academic functioning; similarly, in Saudi Arabia, MD score was associated with poor academic functioning, as high MD score was associated with GPA, putting more emphasis on the tremendous impact of MD on medical students [8].

## Conclusion

MD has shown some alarming rates among University of Khartoum medical students, with more than one-third of total medical students having MD, indicating a serious potential much larger and more pervasive problem. This study should provide a solid foundation for more thorough investigations into the causes and related factors of MD.

## Data Availability

The data that support the findings of this study are available upon request from the corresponding author, Moez M. I. Bashir. The data are not publicly available due to ethical concerns, as they contain information that could compromise the privacy of research participants.

## Abbreviations

MD: Maladaptive daydreaming
MDers: Maladaptive daydreamers
UofK-MED: University of Khartoum, Faculty of Medicine
MDS: Maladaptive Daydreaming Scale
MDS-16-AR: Arabic Version of Maladaptive Daydreaming Scale

## References

[1] Markman KD, Klein WM, Suhr JA (2009) Handbook of Imagination and Mental Simulation. Markman KD, Klein WMP, Suhr JA (ed): Psychology Press, New York http://psycnet.apa.org/record/2008-07500-000.

[2] Singer JL (2014). Daydreaming and fantasy.

[3] Singer JL (1966). Daydreaming.

[4] Bigelsen J, Lehrfeld JM, Jopp DS, Somer E (2016). Maladaptive daydreaming: evidence for an under-researched mental health disorder. Conscious Cogn..

[5] Somer E, Soffer-Dudek N, Ross CA (2017). The comorbidity of daydreaming disorder (maladaptive daydreaming). J Nerv Ment Dis..

[6] Musetti A, Franceschini C, Pingani L, Freda MF, Saita EW, Vegni E, Zenesini C, Quattropani MC, Lenzo V, Margherita G, Lemmo D, Corsano P, Borghi L, Cattivelli R, Plazzi G, Castelnuovo G, Somer E, Schimmenti A (2021) Maladaptive daydreaming in an adult Italian population during the COVID-19 lockdown. Front Psychol 12. 10.3389/fpsyg.2021.631979 10.3389/fpsyg.2021.631979 PMC8024516 33841264

[7] Abu-Rayya HM, Somer E, Meari-Amir S (2019). The psychometric properties of the Arabic 16-item Maladaptive Daydreaming Scale (MDS-16-AR) in a multicountry Arab sample. Psychology of Consciousness: Theory, Research, and Practice.

[8] Alenizi MM, Alenazi SD, Almushir S, Alosaimi A, Alqarni A, Anjum I, Omair A (2020). Impact of maladaptive daydreaming on grade point average (GPA) and the association between maladaptive daydreaming and generalized anxiety disorder (GAD). Cureus.

[9] Somer E, Soffer-Dudek N, Ross CA, Halpern N (2017). Maladaptive daydreaming: proposed diagnostic criteria and their assessment with a structured clinical interview. Psychol Conscious.

[10] Somer E, Lehrfeld J, Jopp DS, Bigelsen J (2016). Development and validation of the Maladaptive Daydreaming Scale (MDS). Conscious Cognition.

[11] Soffer-Dudek N, Somer E (2018). Trapped in a daydream: daily elevations in maladaptive daydreaming are associated with daily psychopathological symptoms. Front Psychiatry.

